# Pilot Randomized Controlled Trial of iCanWork: Theory-Guided Return-to-Work Intervention for Individuals Touched by Cancer

**DOI:** 10.3390/curroncol32050266

**Published:** 2025-05-01

**Authors:** Christine Maheu, Maureen Parkinson, Kyla Johnson, Wing Lam Tock, Naomi Dolgoy, Simon-Pierre Dupuis, Mina Singh

**Affiliations:** 1 Ingram School of Nursing, McGill University, Montréal, QC H3A 2M7, Canada; 2 Vocational Rehabilitation Program, Patient & Family Counselling Services, BC Cancer, Vancouver, BC V5Z 4E6, Canada; mparkins@bccancer.bc.ca; 3 Independent Researcher, Montréal, QC H3T 1E2, Canada; kyla.john@gmail.com; 4 Centre de Recherche du Centre Hospitalier de l’Université de Montréal, Montréal, QC H2X 0A9, Canada; wing.lam.tock@umontreal.ca; 5 Department of Social and Preventive Medicine, École de Santé Publique, Université de Montréal, Montréal, QC H3N 1X9, Canada; 6 Department of Occupational Therapy, University of Alberta, Alberta, AB T6G 2G4, Canada; dolgoy@ualberta.ca; 7 Cancer and Work, Montréal, QC H3A 2M7, Canada; simonpierre@symbiootik.com; 8 School of Nursing, York University, Toronto, ON M3J 1P3, Canada; minsingh@yorku.ca

**Keywords:** return to work (RTW), individuals touched by cancer (ITBC), cancer survivorship, vocational rehabilitation (VR), occupational therapy (OT), work ability index (WAI), feasibility trial

## Abstract

Background: Recent systematic reviews report a limited number of return-to-work (RTW) interventions for individuals touched by cancer (ITBC), with many falling short in effectiveness and lacking an integrated work-health approach. In response, iCanWork—a theoretically informed, multidisciplinary RTW intervention integrating vocational rehabilitation (VR) and occupational therapy (OT)—was conceptualized and developed to address the gap identified in recent reviews for robust, work-health-focused RTW interventions. Methods: A pilot randomized controlled trial was conducted to explore the feasibility, acceptability, and preliminary work-related outcomes of the iCanWork intervention among 23 ITBC participants randomized to either the intervention or control group. Feasibility was assessed through recruitment, retention, and engagement benchmarks; acceptability was measured using a participant satisfaction survey. Preliminary work-health-related outcomes included RTW status, work ability index (WAI) scores, and health-related quality of life (QoL) domains. Results: Feasibility benchmarks were achieved, with 92% recruitment, 83% retention, and 100% completing at least one VR session. Adherence to the session delivery benchmarks was met by 75% of participants before RTW and 41.7% after RTW. Participants rated the intervention highly for its tailored and supportive approach. Compared to the control group, the iCanWork group showed modest improvements in RTW status, WAI scores (mean change: +2.54), and QoL domains, including fatigue, social roles, and pain interference. Given the small sample size, these exploratory findings should be interpreted as preliminary signals to inform outcome selection for a future trial. Conclusions: iCanWork is a feasible and acceptable RTW intervention for ITBC with early indications of benefit. These findings inform the design and outcome selection for a future, larger trial aimed at evaluating the intervention’s potential to improve RTW outcomes for ITBC.

## 1. Introduction

The growing incidence of cancer and improved survival rates present new challenges for the vocational reintegration of individuals touched by cancer (ITBCs) [1]. Throughout this study, we use ITBC to refer to individuals who have experienced a cancer diagnosis at some point in their lives, whether they are in treatment, post-treatment, or navigating life beyond cancer [1].

Returning to work is a crucial step in the healing process for cancer survivors, helping them regain a sense of normalcy and social reintegration [2]. Return to work (RTW) or vocational reintegration refers to the process by which an individual, after a prolonged absence due to health issues, an accident, or other personal reasons, returns to their previous job or transitions into a new job adapted to their current condition [3]. Successful vocational reintegration often involves workplace modifications, reassessment of skills and abilities, and ongoing support to facilitate both return to work and long-term job retention [4].

Despite the employment rate one year after a cancer diagnosis appearing relatively promising—ranging from 52% to 73%—oncology occupational studies reveal a more nuanced reality [5,6]. ITBCs often face negative work outcomes that persist well beyond the first year post-diagnosis, potentially lasting up to a decade [6]. Compared to their healthy counterparts, survivors experience an increased risk of extended sick leaves, resignations, reduced working hours, role changes, and early, unplanned retirement [7,8]. Additional challenges include diminished income, workplace discrimination, and a lack of needed adjustments to work methods. The uncertainty surrounding the chronic illness trajectory can make sustained employment particularly challenging for many survivors.

Addressing the complex and multifaceted challenges of vocational reintegration requires system-level solutions that consider the interplay between individual, organizational, and societal factors. For example, returning to work does not necessarily mean resuming one’s previous workload or work life. Similar to the end of cancer treatment, a return to work does not mark full recovery or a return to previous functional levels. Many ITBCs continue to experience physical, cognitive, and psychological effects following the end of treatment, which can persist for more than a year, such as fatigue and depression, significantly impacting their ability to work and overall job performance after returning to work [6,9,10]. Moreover, stigmatization in the workplace [11] and inadequate employer and colleague support [12], both practical and emotional, serve as major barriers to a successful return to work following a cancer diagnosis. These challenges heighten the risk of job market withdrawal, contributing to higher absenteeism, involuntary resignations, and long-term unemployment among survivors [13]. As a result, ITBCs can experience significant economic losses, with wage reductions estimated between 24% and 43%, depending on employment type [14]. Additionally, household income losses can be compounded by caregiving responsibilities, with associated costs ranging from CAD 15,786 to CAD 20,414 per patient annually, and household productivity losses reaching as high as CAD 238,904 per household per year [14]. The cumulative impact of these barriers leads to substantial financial strain and diminished quality of life for survivors and their families [8].

A comprehensive understanding of systemic factors, and addressing them, is essential for improving the healing process and RTW outcomes for ITBCs. Recent systematic reviews [15,16,17,18] highlight that RTW interventions for ITBCs remain limited in number, and most have shown limited effectiveness. These interventions often fail to adopt a work-health-integrated approach or to be delivered by professionals with specialized expertise in cancer-related vocational rehabilitation (VR), such as VR counselors (VRCs) and occupational therapists (OTs), to address the diverse challenges faced by cancer survivors. VR supports individuals in overcoming employment barriers such as functional challenges, work gaps and the need for accommodation through work-focused counseling, case management from services to improve work function, return-to-work education and planning, and skills training (for example, communicating at the worksite, stress management, and navigating the return-to-work process). Meanwhile, occupational therapists help cancer survivors regain the ability to participate in their valued activities, from daily living to work. OT interventions may include adapting workspaces to accommodate physical limitations, teaching coping strategies for fatigue, or enhancing time management skills. Through personalized strategies, both VR and OT work collaboratively to improve survivors’ functional abilities, quality of life, and overall readiness for workforce reintegration.

ITBCs face significant barriers to accessing multidisciplinary work-focused and rehabilitation supports, particularly due to systemic disparities in access to services. In Canada, ITBCs commonly face workplace stigma, inconsistent employer accommodations, and limited vocational rehabilitation services [5]. Unlike leading European models, where RTW is integrated into oncology care, Canada’s approach remains largely employer-driven, fragmented, and unevenly available across provinces. The absence of nationally structured RTW policies further exacerbates disparities in employment support, often leaving survivors to self-navigate the reintegration process with little formal guidance [19].

The scale of potential demand to reintegrating the workforce following cancer is substantial. In 2024 alone, an estimated 247,100 Canadians were diagnosed with cancer, with over one-third (approximately 91,000) being of working age [20]. Beyond new diagnoses, a much larger population of 555,000 working-age Canadians currently live with or beyond cancer, many of whom experience persistent work-related challenges. Alarmingly, studies estimate that up to 40% of ITBCs face unemployment risks, translating to 222,000 individuals at risk of leaving the workforce that could be prevented if adequate RTW support was available to them [21].

Interventions for oncology vocational rehabilitation work reintegration programs within cancer care centers remain rare and untested. In Canada, only one such program exits [5], yet even this unique initiative does not currently operate in tandem with OT.

In response to the gaps identified by recent Cochrane reviews [16,17], we propose to evaluate a multidimensional, work-health-focused RTW intervention for ITBCs called iCanWork. Developed through the extensive experience of our multidisciplinary team, whose contributions include randomized controlled trials (RCTs) with ITBCs and the creation of nationally recognized RTW resources (e.g., www.cancerandwork.ca), the iCanWork intervention is proposed as an online, one-on-one, professionally led, multidisciplinary intervention that brings together VR counseling and OT support.

**Figure 1:**
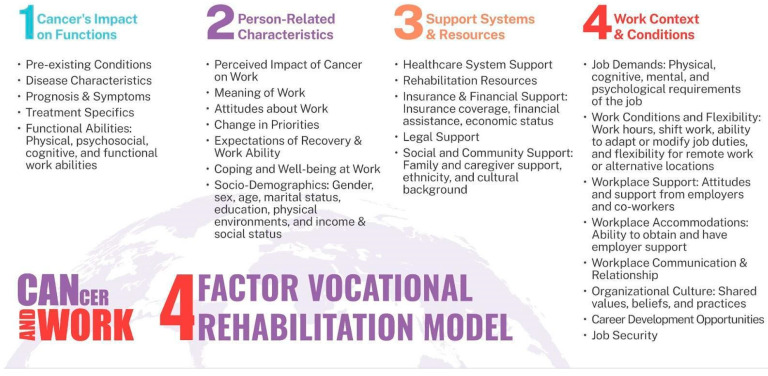
Cancer and work: four factors vocational rehabilitation model for individuals living with cancer. Note: Revised from the original model, “Vocational Rehabilitation Model for Cancer Survivors” [22].

The iCanWork intervention is a structured VR program designed to support ITBCs in their RTW journey. The iCanWork intervention is grounded in the four-factor VR model for ITBCs (See Figure 1) [22,23,24]. The four-factor VR model for ITBCs addresses the multifaceted relationship between the impact of cancer on person-related functions and the characteristics of system factors, and work context and conditions that influence the RTW process. The first factor, Cancer’s Impact on Functions, focuses on the effects of cancer, its treatments, and pre-existing conditions, on physical, psychological, and cognitive functions, highlighting areas that may present challenges to RTW efforts. The second factor, Person-Related Characteristics, explores individual attitudes towards work and recovery while considering socio-demographic elements such as age, gender, and income that shape the reintegration process. The third factor, Support Systems and Resources, evaluates the availability and effectiveness of healthcare/rehabilitation services, insurance, and social support systems that are essential for maintaining and resuming employment. Finally, the fourth factor, Work Context and Conditions, examines job demands, workplace accommodations, and organizational culture to assess how these factors align with the individual’s work access after cancer treatment.

This study aimed to pilot-test the iCanWork intervention through a randomized controlled feasibility trial. Specifically, the objectives were (a) to explore the feasibility of the intervention and (b) its acceptability as a RTW approach, and (c) to generate preliminary data on key cancer and work outcomes (RTW status, work ability, and health-related quality of life) to inform the selection of a primary outcome for a future definitive trial.

## 2. Materials and Methods

### 2.1. Study and Intervention Design

This multicenter pilot randomized controlled trial (RCT) employed a 1:1 parallel group design, comparing an intervention group to a control group. This study followed the guidelines set forth by the Consolidated Standards of Reporting Trials (CONSORT) 2010 for randomized pilot and feasibility trials (Supplementary Table S1) [25], ensuring a structured and transparent reporting process. Additional guidance was drawn from the Template for Intervention Description and Replication (TIDieR) (Supplementary Table S2) [26], which provides a framework for describing interventions in detail. This study received ethical approval from the Research Ethics Boards of the first author, with primary clinical sites located in Montreal, Quebec.

The iCanWork intervention was designed to operationalize the four-factor VR model into practical strategies that address the unique challenges faced by ITBCs. As detailed in Supplementary Table S3, this tailored approach integrates specific assessment tools and intervention strategies that align with participants’ identified needs and their likelihood to RTW and sustain employment (categorized as high, moderate, or low), as determined by the CAWSE at baseline and during follow-up sessions [1]. Participants are classified based on their likelihood to RTW, and specific CAWSE items with low scores help identify the individual challenges requiring targeted interventions.

As illustrated in Supplementary Table S3 under “Example Thesaurus, e”, when participants score low on the coping and well-being at work subscale, they are offered strategies and resources to enhance psychological support, such as cognitive–behavioral strategies or mindfulness techniques, to promote emotional well-being and improve coping. Similarly, those who report fatigue, memory difficulties, or concentration challenges—particularly within the perceived impact of cancer on work subscale—receive tailored support such as energy management training, cognitive rehabilitation referrals, or workplace accommodation guidance to facilitate sustained employment.

In addition, ITBCs who scored low on the CAWSE subscale for Support, Communication, and Accommodations at Work would be provided with guidance on improving communication strategies with employers, with referrals to workplace mediation services, or strategies for advocating flexible work arrangements. As described in Supplementary Table S3 (components FU1, FU2, FU3), follow-up sessions are structured to evaluate participants’ progress, adjust the intervention based on persisting barriers, and assess the readiness for sustained employment. For ITBCs with low scores on the CAWSE financial and insurance support subscale, interventions include connections to financial planning resources or education on employment rights. Those reporting social pressure or workplace stigma—identified through low scores on the CAWSE subscale workplace, economic, and external factors—are offered support to enhance social inclusion and workplace advocacy.

In addition to the CAWSE, the intervention incorporates several validated tools to guide tailored support strategies. These tools are used to inform care rather than as outcome measures. As outlined in Supplementary Table S3 (components b-e), these tools support the intervention adjustments throughout ITBC engagement with iCanWork. For example, the work ability index (WAI) [27] helps assess participants’ capacity to meet the physical, cognitive, and emotional demands of their job, relative to their current health status and mental resources [28], while the multidimensional work motivation scale (MWMS) [29] evaluates various dimensions of work motivation. Psychological well-being is assessed using MoodFx [30], a screening tool for symptoms of depression, anxiety, and cognitive challenges, alongside the PHQ-9 [31] for depression, and generalized anxiety disorder 7 (GAD-7) [32] for general anxiety. Cognitive challenges affecting work performance are identified through the occupational cognitive failures questionnaire (OCFQ) [33], and the cancer and work cognitive symptoms at work checklist [34]. Barriers to accessing healthcare services are explored using the barriers to access to care evaluation (BACE) [35], while workplace support is assessed with the perceived organizational support (POS) scale [36]. Lastly, participants’ flexibility, resilience, and career adaptability are evaluated using the career adapt-abilities scale [37].

Importantly, while these assessment tools were essential in tailoring the iCanWork intervention to participants’ specific needs, they were not included as primary or secondary study outcomes. Rather, these measures informed individualized intervention strategies by identifying areas that required additional support. This study’s primary and secondary outcomes focused on assessing the intervention’s feasibility and acceptability, while also exploring distal preliminary efficacy to help identify a potential primary outcome for a larger trial.

The development of iCanWork followed an iterative and evidence-informed process, beginning in 2011 with the publication of Cancer and Returning to Work: A Practical Guide for Cancer Patients [38]. This workbook, authored by co-author M. Parkinson, a master’s-level oncology VR counselor with 30 years of experience in cancer-related RTW interventions, was evaluated by 60 experts with professional and lived experience, including ILWCs and employer representatives. Recognized as a critical resource for clinical practice, the workbook laid the foundation for the creation of the Cancer and Work website, launched in 2016 [23]. This comprehensive 600-page platform provides evidence-based information, tools, and resources to support RTW. One of its key offerings, the iCanWork 10 Step RTW Intervention, delivers step-by-step guidance for ILWCs, healthcare providers, and employers to navigate the RTW process effectively.

Before launching the Cancer and Work website, our team conducted an acceptability evaluation of its content and resources. Feedback from 250 users, including ILWCs and healthcare professionals, highlighted the comprehensiveness, completeness, and usefulness of both the website and the iCanWork 10 Step RTW Intervention, though they also noted its complexity [22]. A second usability review in 2019 corroborated these findings and included a key recommendation to enhance the iCanWork 10 Steps RTW Intervention with online support to increase accessibility and user engagement [22,39].

To maximize the potential efficacy of the enhanced iCanWork 10 Steps RTW Intervention for ILWC, we have incorporated the latest recommendations from systematic reviews to integrate VR and OT expertise into the resource and theoretically ground it in the four-factor VR model for ITBCs [22,40]. This integration aims to provide a multidisciplinary approach that addresses the unique challenges faced by ILWC during their RTW process. Notably, a VR/OT approach has not yet been adequately explored in this context, underscoring the innovative and necessary nature of this intervention [15,41,42].

### 2.2. Setting, Recruitment, Participants, and Sample Size

The participants of this study were recruited from ambulatory follow-up cancer care clinics at two participating cancer centers in Montreal, Quebec, through referrals from oncologists and nurses. Additionally, approximately half of the participants self-referred in response to advertisements placed on the Cancer and Work website and through Quebec-based cancer organizations, such as the Quebec Cancer Foundation, from May 2022 to August 2022.

Eligibility criteria included individuals who (1) had been diagnosed with any type of cancer treated with curative intent and of working age (between 18 and 65 years at the time of diagnosis); (2) were employed in a paid position at the time of diagnosis (full-time or part-time) and have the intent to return to the same position; (3) were on cancer-related sick leave, including long-term disability leave, but for less than two years; (4) had not initiated a RTW, including partial RTW, since their leave due to cancer; (5) were able to read and understand English or French; and (6) resided in the province of Quebec. The inclusion criterion requiring participants to reside in Quebec was necessary because the work-focused health professionals delivering the intervention were licensed in the province of Quebec.

The sample size for this external pilot RCT was determined based on this study’s primary aim: to assess the feasibility and acceptability of the iCanWork intervention and generate preliminary data on key cancer and work outcomes to inform the design of a future definitive trial. In line with guidance from Eldridge et al. [25] and Lancaster et al. [43], the sample size was not based on hypothesis testing, but rather on the need to estimate key feasibility parameters such as recruitment, retention, and intervention delivery. Meanwhile, simulation studies have suggested that estimating outcome variance with high precision may require larger samples in the range of 60–70 participants [43,44,45,46]. Given that this study was not designed to estimate outcome variance or to conduct hypothesis testing, no formal power calculation for sample size was conducted. Instead, based on recommendations for pilot RCTs, a minimum target sample size of 12 participants per group was established [45].

### 2.3. Randomization and Blinding

The study coordinator scheduled the initial appointment with eligible participants via videoconferencing. During this session, the participants received a detailed explanation of this study’s purpose, followed by a question-and-answer period to address any concerns. Upon obtaining informed electronic consent, the participants proceeded to complete baseline assessments (T0), which included demographic and medical information as well as the designated self-reported study questionnaires. Once baseline data collection was completed, the study coordinator accessed a secure, single-use locked folder to determine the participant’s allocation to either the iCanWork intervention or the control arm, using a 1:1 randomization ratio. The randomization sequence was pre-generated by a statistician at the Research Institute of the McGill University Health Centre (RI-MUHC) and securely maintained. To ensure allocation concealment and minimize potential bias, the sequence remained blinded to both the study coordinator and participants, thereby guaranteeing an impartial assignment process.

### 2.4. Study Groups

Intervention Group: Along with being introduced and referred to the Cancer and Work website for information on RTW after cancer, participants in the intervention group were scheduled to receive professional VR and OT support. Once enrolled and randomized to the iCanWork intervention group, the study coordinator scheduled their first session with this study’s VR counselor. The iCanWork RTW intervention was delivered via individual videoconferencing and recorded to ensure fidelity in the delivery of the intervention. Fidelity was reviewed by CM and MP for all sessions, with a specific focus on the first session, which was reviewed within 24 h of its delivery. Recommendations for session delivery improvements were made by CM and MP during a videoconference within the first week of the session date, and these recommendations were communicated to the VR counselor or OT responsible for delivering the session to enhance consistency and adherence to the intervention protocol.

Participants received between one and three VR counseling sessions and, if deemed clinically needed as per the VR assessment, participants could receive one to two OT sessions. These sessions were tailored to individual needs, with the first session lasting approximately 1.5 h and subsequent sessions lasting an hour each. The number of sessions provided was constrained by funding limitations, though in practice, more sessions might be required.

The intervention emphasized flexibility to accommodate participants’ preferences, offering sessions via telephone for those uncomfortable with videoconferencing, scheduled at a time convenient for them. Each session concluded with a written summary of recommendations, which was sent to the participant within 24 h. Supplementary document S4 includes examples of intake sessions and summary notes: an initial vocational rehabilitation assessment session with recommendations shared with the participant, an occupational therapist intake session with corresponding summary notes, and an example of a follow-up session conducted by this study’s vocational rehabilitation counselor.

Control Group: Participants in the control group were directed to the Cancer and Work website (www.cancerandwork.ca) [23], a comprehensive, bilingual and educational platform that offers evidence-informed tools and information to support RTW after cancer. While the website serves as a valuable resource for self-guided learning and planning, it does not provide direct clinical services such as VR counseling or OT sessions. Control group participants were instructed to download a bilingual pamphlet summarizing the website’s key content. They were also encouraged to consult their healthcare team for any additional RTW-related concerns or guidance.

The choice of control group conditions were informed from Cochrane [15,16] and scoping reviews [47] that identified key considerations for RTW and cancer study control groups. Our design controlled for general exposure to cancer-related information resources by ensuring that both groups were aware and knew where to access the Cancer and Work website. However, the control condition lacks dedicated RTW guidance, individualized counseling, and employer communication strategies—core elements of the iCanWork intervention. Thus, our design controls for passive access to information but does not control for active professional RTW guidance from VR and OT, key essentials for improving employment outcomes [15,16,47]. Any additional RTW-related services accessed by control group participants, such as ad hoc VR or OT support, were collected at the second study measure three months after baseline (T2), documented during follow-ups. However, as previously noted, structured multidisciplinary involvement of VR counselors and OT professionals in Canadian cancer care centers is virtually non-existent. Only one Canadian cancer center is known to offer VR services, and even then, this is provided as a standalone service rather than as part of an integrated cancer survivorship multidisciplinary team [5].

### 2.5. Data Collection

Data collection took place over a six-month period during the summer of 2022. Potential participants were first emailed a personalized email link directing them to an electronic consent form hosted on Qualtrics. This form included a detailed study description and the IRB-approved consent statement. The participants provided informed consent by submitting their electronic signature. Upon completion of the consent form, the baseline questionnaire package (T1) was released to participants via Qualtrics.

The T1 questionnaire included demographic and clinical information, as well as all baseline study outcome measures. The same set of outcome measures—excluding demographic and clinical questions—was administered again at three months post-baseline (T2), using the same Qualtrics platform and email-based distribution method. The T2 package also included a satisfaction questionnaire for participants in both the intervention and control groups. Completion of each survey package took less than 30 min.

Additionally, at six months post-enrollment, qualitative feedback was collected from participants in the iCanWork intervention group through a single open-ended question: “In what ways, if any, did the support received from the iCanWork intervention help you with RTW?” This question was also distributed via email using a Qualtrics link.

Data collection logs were maintained throughout this study to monitor completion and ensure fidelity to study procedures. Follow-up reminders and technical assistance were provided by the research assistant to maximize response rates and ensure data completeness.

### 2.6. Measurements of Study Outcomes

**Figure 2:**
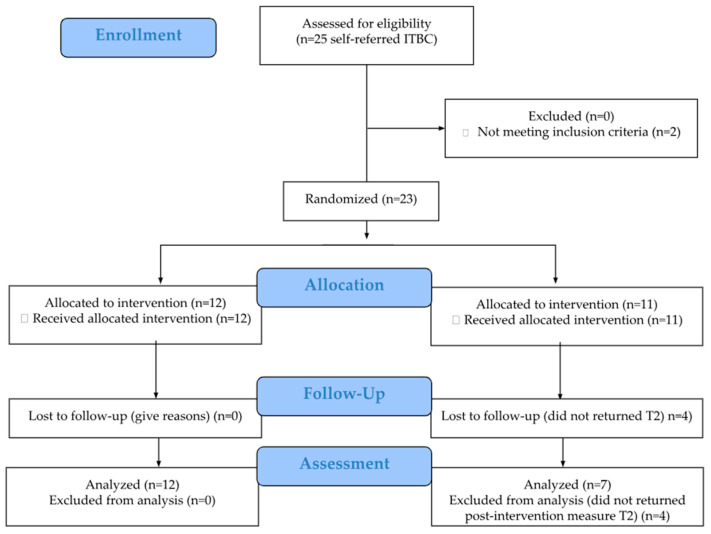
CONSORT flow diagram.

Feasibility was evaluated using recruitment, retention, and intervention fidelity rates, benchmarked against established thresholds for pilot studies. The specific benchmarks were as follows: (i) achieving a recruitment rate of at least 50% at three months of enrollment opening, (ii) maintaining a retention rate of at least 70%, and (iii) participant engagement with the intervention, defined as completing at least one VR session. In addition, two session delivery adherence benchmarks were set: (a) before RTW adherence, defined as completing at least one VR session and, if deemed necessary, one OT session prior to RTW; and (b) after RTW adherence, defined as completing at least one VR session and one OT session, if needed, following RTW. These feasibility outcomes align with the Consolidated Standards of Reporting Trials (CONSORT) requirements for clinical trials in eHealth (CONSORT-EHEALTH v1.6.1) [48,49]. As shown in the flow diagram in Figure 2, the log documented recruitment and retention rates, including the number of patients approached, self-referred participants, eligible and ineligible patients, reasons for ineligibility, participants declining participation (with reasons), and those who consented and were randomized.

To optimize intervention fidelity, we aimed to have 90% of sessions conducted by the VR counselor or OT to be reviewed by the two principal study leads within 48 h of completion [50]. This review process was implemented as an additional benchmark for maintaining consistency and adherence to the intervention protocol, ensuring high-quality delivery of the iCanWork intervention across all participants.

Acceptability was assessed using the Satisfaction with Therapy and Therapist Scale-Revised (STTS-R) [51,52], a 13-item questionnaire adapted to evaluate satisfaction with the iCanWork intervention and its delivery. Participants responded on a 5-point Likert scale ranging from strongly disagree (1) to strongly agree (5), with higher scores indicating greater satisfaction. Three key satisfaction domains are evaluated: satisfaction with the intervention, satisfaction with the therapist delivering the intervention, and perceived changes in the participant’s condition. Higher scores indicate greater satisfaction and a more positive endorsement of the intervention. Recognized for its robust psychometric properties [52], the STTS-R remains a validated and reliable tool for assessing therapeutic acceptability. The STTS-R has also been used successfully in studies evaluating internet-based interventions [53,54]. To ensure consistency in assessing satisfaction across both study groups, the STTS-R was administered to participants in both the intervention and control groups at three months post-baseline (T2). For control group participants, the Cancer and Work website, its chatbot, and the research assistant’s email guidance collectively served as the intended intervention and interventionist equivalent.

Study Outcomes: Two work-related outcomes and one clinical outcome were evaluated. The first work outcome—RTW status—was assessed using a binary (Yes/No) item asking “Are you back to work following your sick leave for cancer, in any form—part-time, full-time, or progressive return to work?” [55,56,57]. In addition to binary RTW status for measuring a work outcome, work ability was also assessed using the work ability index (WAI) [27]. The WAI is the most widely used questionnaire for the self-assessment of work ability [58]. The WAI is designed to evaluate a worker’s capacity to meet job demands while considering their health status and mental resources [1]. The WAI includes seven dimensions that collectively assess an individual’s overall work ability. These dimensions include current work ability compared to lifetime best (score range: 0–10); work ability in relation to job demands (score range: 2–10); number of physician-diagnosed diseases (score range: 1–7); estimated work impairment due to illness (score range: 1–6); absenteeism in the past 12 months (score range: 1–5); personal prognosis of work ability in two years (score range: 1–7); and mental resources (score range: 1–4).

The WAI total score ranges from 7 to 49 points, with higher scores indicating better work ability. Standard WAI categories are defined as Excellent (44–49), Good (37–43), Moderate (28–36), and Poor (7–27). Meaningful changes in WAI scores are typically interpreted as follows: an improvement of 2 to 3 points is considered clinically relevant, reflecting enhanced work ability. The WAI has demonstrated strong construct validity (r = 0.72 with SF-36) and satisfactory internal consistency (Cronbach’s alpha = 0.72) [59]. Furthermore, the WAI has been found to reliably predict work disability, retirement, and mortality [60].

The clinical study outcome quality of life (QoL) was measured using the Patient-Reported Outcomes Measurement Information System (PROMIS)-29 adult profile [61]. PROMIS-29 is validated in cancer populations [62] and assesses QoL across eight health domains: physical function, anxiety, depression, fatigue, sleep disturbance, ability to participate in social roles and activities, pain interference, and pain intensity. These domains are particularly relevant as they are known to influence the capacity for sustained employment in ITBC [63,64]. All domains were retained in the analysis, as the iCanWork intervention targets multiple barriers to RTW, including emotional distress, fatigue, and reduced functional and social role capacity.

Unlike cancer-specific tools such as the EORTC QLQ-C30 [65], PROMIS-29 offers streamlined scoring and allows for cross-population comparisons using domain-level T-scores benchmarked to general population norms. A recent empirical comparison between PROMIS-29 and the EORTC QLQ-C30 in a large cancer cohort demonstrated strong conceptual and statistical agreement between the instruments, supporting their comparability and interchangeable use for QoL assessment in cancer care [66].

### 2.7. Data Analysis

Consistent with the aims of a pilot feasibility trial, analyses were primarily descriptive, focusing on feasibility, acceptability, and generating preliminary data on return-to-work status, work ability, and quality of life to inform the selection of a primary outcome for a future definitive trial. Descriptive statistics (means, medians, standard deviations, frequencies, and percentages) were used to summarize participant demographics, clinical characteristics, and intervention engagement metrics. These data analysis approaches are consistent with those commonly used in return-to-work (RTW) pilot feasibility trials among cancer survivors [67,68].

Feasibility outcomes—such as recruitment, retention, and adherence—were summarized descriptively and evaluated against pre-established feasibility benchmarks. Acceptability was assessed by calculating the proportion of high satisfaction ratings (scores of 4 or 5 on a 5-point Likert scale) across STTS-R items [52].

Work-Health Related Outcomes Analysis—RTW status was analyzed both as a binary variable (returned versus not returned to work, as measured at T2) and as a time-to-event outcome [67,68]. Kaplan–Meier survival analysis was used to explore differences in time to RTW between the intervention and control groups, with group comparisons assessed using the Log-Rank tests [60,64]. Median and mean time to RTW were calculated descriptively for each group. Given the small sample size and the exploratory nature of this pilot trial, these analyses were interpreted descriptively to inform outcome selection and the design of a future definitive trial.

Due to small cell sizes (fewer than five observations in some categories), Fisher’s Exact Test was used to compare RTW proportions and WAI categories between groups. WAI scores were categorized into predefined action categories (Poor, Moderate, Good, Excellent) [25], and differences in categorical distributions were explored.

WAI and QoL scores were additionally analyzed as continuous outcomes. Given the small sample size, changes in WAI and QoL scores from T1 to T2 were assessed using non-parametric tests. Wilcoxon Signed Rank tests were used for within-group changes, and Mann–Whitney U tests for between-group comparisons. All inferential tests were interpreted descriptively, and no adjustments were made for multiple comparisons. All data analyses were conducted using IBM SPSS Statistics (v26).

## 3. Results

### 3.1. Participant Characteristics

A total of 23 participants were randomized, with 12 assigned to the iCanWork intervention group and 11 to the control group (Figure 2). The mean age was 45.6 years (SD = 9.01), with the majority being female (95.7%) and having completed higher education (78.2%). Breast cancer was the most common diagnosis (82.6%), and 78.3% of participants underwent chemotherapy. The mean duration from work leave to enrollment was 11.1 months (iCanWork = 9.75 months, Control = 12.5 months). The National Occupational Classification (NOC) is a system that identifies and categorizes jobs through numbered codes [69].

Participants were employed across NOC-categorized occupations, including the following: professional health services (n = 2); education, teaching, and law (n = 5); and business, including human resources, advertising, and management (n = 16). No significant baseline differences in demographics or study variables were observed, except for Physical Function and Pain Interference, which were controlled for in subsequent analyses. Table 1 provides a full detailed description of the study sample.

### 3.2. Feasibility

The feasibility benchmarks demonstrated strong outcomes across multiple metrics. The target sample size was 12 participants per group; by the end of the recruitment period, 12 participants were enrolled in the intervention group, and 11 were enrolled in the control group. Of the 25 self-referred participants, 23 met the inclusion criteria, agreed to participate, completed the baseline measure (T1), and were randomized, yielding a recruitment rate of 92%, which exceeded the pre-specified benchmark of 50% at three months [70].

Retention was defined by completion of the T2 questionnaire and reached 83% overall. Specifically, all intervention participants (100%) and 63.6% (7/11) of control participants completed the post-intervention T2 measures, surpassing the 70% benchmark.

Intervention fidelity was assessed through independent review of session recordings by the first two co-authors. Both the VR counselor and the OT received training in the iCanWork 10-step intervention and successfully completed the accredited module of this intervention [71]. The OT had over 20 years of oncology experience. Fidelity review confirmed that 90% of session content was delivered as intended (see Supplementary Table S3), indicating strong adherence to intervention protocols [70].

Regarding data completeness, the intervention group had no missing questionnaire data. The control group, however, showed a 23% rate of missing data in the T2 questionnaire, exceeding the 10% threshold for acceptable missing data [70]. 

#### Participant Engagement and Benchmark Adherence

Table 2 summarizes participant engagement and adherence to intervention delivery benchmarks, focusing on VR and OT sessions. Engagement was defined as receiving at least one VR session. Before RTW, adherence required at least one VR session and, if indicated, one OT session prior to RTW. After RTW, benchmark required at least one VR session and one OT session, if needed, following RTW.

In total, 75% (9/12) of participants met the before-RTW benchmark, and 41.7% of participants (5/12) met the after-RTW benchmark. Barriers to meeting after-RTW benchmarks included scheduling conflicts, delayed RTW attempts, and study conclusion prior to session delivery. Participants P11 and P12 expressed interest in additional VR sessions but were unable to complete them before this study closed. 

### 3.3. Acceptability of the iCanWork Intervention

Participants reported high satisfaction with the iCanWork intervention, particularly highlighting the interventionist’s listening skills, warmth, and non-critical attitude. On the Satisfaction with Therapist Scale (STTS-R) [51,52], 100% of participants agreed or strongly agreed with several items (Items 2, 4, 8, 10) (see Table 3 for full results). Overall satisfaction with the intervention, as measured by the Satisfaction with Therapy Scale (Item 1), was 75.0%, and 83.3% agreed that it helped address their specific problems (Item 13).

Satisfaction ratings in the control group were generally lower than those in the intervention group, with only 5 of the 11 participants providing responses (Table 3). As described in the methods, the Cancer and Work website, the chatbot, and the research assistant’s email guidance collectively served as the intended intervention and interventionist equivalent for control group participants. This design may explain the control group’s engagement patterns and lower satisfaction ratings, or their interpretation of certain STTS-R items. As such, only two of the five participants reported being satisfied with the intervention (Item 1) and agreed that the interventionist provided an adequate explanation (Item 4). However, despite these lower proportions of satisfaction for the intervention, control group participants reported levels of agreement similar to the intervention group when asked how much the intervention helped with the specific problem that led them to enroll in the program (Item 13), suggesting that some participants found value in the educational content despite limited engagement.

At six months post-enrollment, feedback was collected from the twelve participants in the iCanWork intervention through an open-ended question: “In what ways, if any, did the support received from the iCanWork intervention help you with your return to work?” Participants highlighted key areas where the intervention had a positive impact. Many reported feeling more confident in their ability to re-enter the workforce, attributing this to the program’s tailored support and guidance. Improved communication skills were also noted, as participants felt better equipped to articulate their needs and limitations to employers and colleagues. The program was described as instrumental in facilitating discussions about workplace accommodations, which contributed to smoother transitions back to work. Emotional support from the interventionist was frequently mentioned, helping participants manage anxiety and fear associated with returning to work. Additionally, practical strategies for managing workload and fatigue were highlighted as essential tools that enabled participants to navigate their return successfully. These qualitative insights illustrate the comprehensive benefits of the iCanWork intervention in addressing the complex challenges faced by ITBCs in the process of returning to work.

### 3.4. Preliminary Work-Health Related Outcomes

#### Return to Work Status

Table 4 presents the RTW status and time to RTW by group allocation. At T2, 50% of participants in the iCanWork group had returned to full-time work compared to 36.4% in the control group. Part-time RTW was reported in 33.3% and 54.5% of participants, respectively, while 16.7% of the iCanWork group and 9.1% of the control group had not RTW.

Median time to RTW was 8 months for the iCanWork group and 11 months for the control group, with corresponding means of 10.4 and 13.5 months. While Fisher’s Exact Test (*p* = 1.000) and Kaplan–Meier survival analysis with Log-rank test *p* = 0.313 were used to explore group differences, these comparisons are presented for descriptive purposes only. Given the small sample size and the high degree of missing data in the control group, *p*-values should be interpreted with caution, and findings should not be considered statistically conclusive.

### 3.5. Work Ability Index (WAI)

Non-parametric Mann–Whitney U tests indicated that the change in WAI scores from T1 to T2 did not differ significantly between the intervention and control groups.

**Figure 3:**
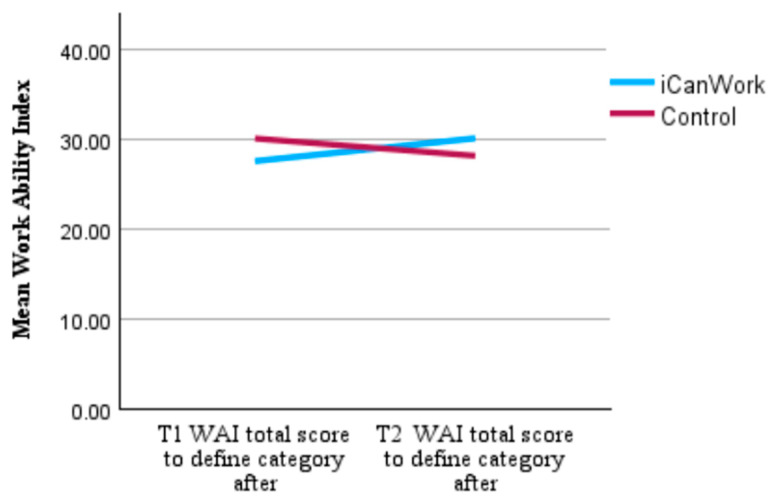
Mean WAI score at T1 and T2 (higher score indicates better work functioning).

Figure 3 presents a descriptive comparison of group means across time, showing a slight increase in WAI scores for the iCanWork group from T1 to T2 (27.63→30.17), and a slight decrease for the control group (30.12→28.21).

To better contextualize these changes, participants’ WAI scores were categorized into the standard categories of Excellent, Good, Moderate, and Poor. Despite some observed score shifts, no statistically significant differences emerged between groups. The distribution of participants in both groups falling within the Poor category was 44.4% in iCanWork vs. 55.6% in Control at T1. By T2, the proportion of participants in the Poor category increased to 71.4% in the iCanWork group, but decreased to 28.6% in the Control group (Table 5). Additionally, individual-level changes in WAI scores revealed a mixed pattern of changes across both groups. In the iCanWork group, 50% of participants (6/12) demonstrated score improvements, while five participants experienced declines, and one participant remained unchanged. A similar trend was observed in the control group, where three participants improved, three showed declines, and one remained unchanged. Despite these observations, the results highlight persistent work ability challenges in both groups over the three-month period.

### 3.6. PROPr QoL Domains

Table 6 presents a detailed overview of baseline (T1) and post-three month (T2) PROPr QoL across the eight domains for both study groups. Mann–Whitney U results indicate differences in scores whereby the iCanWork group had significantly improved scores for fatigue (*p* = 0.040), social roles and activities (*p* = 0.017), and pain interference (*p* = 0.006). No significant improvements were noted for the other five QoL domains. Given the small sample size and the feasibility focus of this study, these comparisons are considered exploratory and should be interpreted as preliminary signals to inform outcome selection for a future definitive trial.

### 3.7. Specific Support Services Utilized Post-Consultation

Following their consultation, participants were recommended specific support services to address work functioning challenges and the impact of cancer and its treatment, including cancer-related fatigue and cognitive changes. These recommendations included physiotherapy and structured exercise programs for fatigue management, as well as referrals to physicians to investigate potential underlying biological causes. For cognitive changes, participants were advised to seek comprehensive assessments from a neuropsychologist or occupational therapist. At three months after baseline measure, 10 out of 12 participants in the intervention group reported utilizing services such as physiotherapy, OT, and osteopathy.

## 4. Discussion

This pilot randomized controlled trial was conducted to explore the feasibility and acceptability of iCanWork, a theoretically informed, multidisciplinary RTW intervention for ITBCs. In line with the aims of a feasibility trial, this study also sought to generate preliminary data on key work-health outcomes—RTW status, work ability, and quality of life—to inform the selection of a primary outcome for a future definitive trial. The intervention was developed in response to a well-documented gap in oncology care models, which rarely integrate VR and OT to address the complex challenges faced by ITBCs in their RTW process.

Among the 23 participants enrolled—12 in the intervention group and 11 in the control group—this study demonstrated strong feasibility, exceeding established recruitment (92%) and retention (83%) benchmarks. The intervention was delivered with high fidelity, and the participants reported high levels of satisfaction with the tailored support provided by the VR and OT professionals.

Preliminary outcome data showed descriptive trends favoring the intervention group. At three months post-enrollment, 50% of participants in the iCanWork group had returned to full-time work, compared to 36.4% in the control group. Median time to RTW was also shorter in the intervention group (8 months vs. 11 months). While these differences were not statistically significant, the findings—based on a small sample size and exploratory pilot RCT design—are consistent with broader trends suggesting that professionally delivered support may positively influence RTW outcomes for ITBCs [72,73,74].

Similar trends were observed for WAI and QoL, with modest improvements observed only in the iCanWork group. Although no statistically significant differences were found, the direction of effects and participant-reported benefits suggest potential value in the intervention, warranting further evaluation in a fully powered trial.

### 4.1. Comparison of iCanWork with Existing RTW Support Interventions

RTW interventions for ITBCs vary widely in their structure, approach, and targeted outcomes [16]. The iCanWork intervention is distinctive in its integration of VR and OT within a flexible, person-centered framework that focuses on supporting individuals rather than employers directly. Unlike employer-focused models such as MiLES [75], which aim to educate and guide employers, iCanWork equips ITBCs with the tools and strategies they need to manage their RTW journey. This includes consulting with participants on how to communicate their workplace needs, request accommodations, and actively engage employers in the RTW planning process, when possible. While employers may become involved as a result of these participant-led efforts, iCanWork remains fundamentally a person-centered intervention for ITBCs. In contrast to the Beyond Cancer program [73,74], which delivers long-term, individualized multimodal rehabilitation primarily through occupational rehabilitation consultants—with employer engagement offered but not routinely implemented—the iCanWork intervention provides a structured, theory-informed, and multidisciplinary approach. Specifically, iCanWork integrates VR counseling with OT, incorporating systematic worksite assessments and individualized accommodation planning. This structured integration ensures that return-to-work strategies are directly aligned with the survivor’s job demands and functional abilities, offering a more targeted and comprehensive pathway to sustainable employment outcomes. Similarly, while Leensen et al. [72] combine occupational counseling with physical exercise, their study lacks the structured vocational assessment and workplace accommodations planning that iCanWork delivers.

A major strength of iCanWork is its multidisciplinary engagement, incorporating VR specialists, OTs, and work context and conditions data to comprehensively guide the RTW of ITBCs. A recent Cochrane review exploring non-medical interventions for RTW in cancer populations concluded that future research exploring multidisciplinary approaches and VR interventions are warranted [16]. The inclusion of both OT and VR in iCanWork ensures that the individual and their work needs are explored in connection with their functional needs as a whole. Having a whole-person functional perspective within an RTW-focused cancer program targets the specifics of work with attention to functional engagement and practical application [76]. The benefit of iCanWork is that programming supports individuals across all RTW stages, whereas Zovacevic et al. [77] emphasizes only early prehabilitation and vocational reintegration, and Zaman et al. [78] primarily provide early work-related support before treatment. Consideration of the individual in context of their functional needs, as offered through the inclusion of oncology OT and VR, provides a mechanism for program success and sustainment, being adaptable to tailored interventions and changing individual functional capabilities.

### 4.2. Areas for Enhancement for iCanWork Based on Specific Components of Other RTW Interventions

While iCanWork presents several advantages, insights from our findings can utilize components from other interventions to enhance areas in further future iCanWork research and intervention development. One area of improvement that was found to be limited in our post-pilot data is employer engagement. The MiLES program [75] employs a web-based employer toolkit for workplace accommodations and phased RTW planning. Future iterations and expansion of iCanWork could benefit from developing an employer resource package to facilitate better workplace support, integration, and communication.

Another important area for enhancement found post-pilot is the need for the integration of structured physical rehabilitation. Leensen et al. [70] offers examples of how structured physical activity programs can improve RTW outcomes of cancer patients; further, the combination of multidisciplinary approaches has been called for given the sparse research specific to VR [16]. Adding tailored exercise regimens or partnerships with physiotherapists could enhance iCanWork outcomes in future research with respect to fatigue management and physical function [79]. Although the VR and OT professionals delivering iCanWork actively encourage participants to engage in physical activity and seek appropriate referrals, further strengthening of these elements will likely optimize intervention outcomes. Specifically, participants are encouraged to consult a physiotherapist for a comprehensive assessment of physical limitations and rehabilitation needs, following obtaining medical clearance that engaging in physical activity is safe. Additionally, referrals to a kinesiologist could assist in developing a structured physical retraining program when necessary [16]. These targeted efforts would ensure that physical reconditioning is addressed as a core component of the RTW process, potentially mitigating cancer-related fatigue and improving functional capacity, thereby facilitating sustained workforce reintegration. By embedding structured physical rehabilitation within iCanWork, the intervention could provide a more holistic approach to addressing the multifaceted barriers cancer survivors face in returning to work.

Current iCanWork findings do not address prehabilitation and early RTW screenings. Utilizing the findings of Zaman et al. [78], iCanWork could enhance future iterations to include earlier intervention before treatment, such as prehabilitation assessments to identify work-related challenges early and develop pre-treatment vocational strategies to ensure a smoother reintegration process [80].

Finally, iCanWork post-pilot findings point to the need to enhance future outcomes with respect to digital and remote RTW support. Again, exploring MiLES [75] and Beyond Cancer [73] offers examples of enhancement opportunities through the use of digital technology and web-based interventions. While current iCanWork programming includes a telehealth model integrating VR and OT, future iterations could expand on digital approaches through the integration of a digital component, such as a mobile application, to enhance intervention engagement by enabling participants to self-track their work ability, monitor progress, and receive tailored recommendations based on their evolving needs. This innovation would not only improve scalability, but also foster greater participant autonomy in managing their RTW journey while maintaining the high level of personalized support that is integral to iCanWork’s success.

The limited availability of oncology-specific VR tools posed challenges in translating some iCanWork findings into practical clinical applications. While our selected assessment instruments effectively informed individualized intervention strategies, they were not primarily designed to evaluate RTW outcomes directly. Future iterations of iCanWork research and programming could benefit from utilizing the validated CAWSE [1] as a central work outcome measure in ITBCs. Additionally, incorporating measures of self-efficacy and psychological distress is recommended, as these factors have been shown to significantly impact RTW outcomes among cancer survivors. Research indicates that higher self-efficacy is associated with improved RTW rates [81], while elevated psychological distress can delay and hinder successful reintegration into the workplace [82,83]. Additionally, studies have demonstrated that psychological distress, fatigue, pain, and cognitive impairments are prominent barriers to RTW in cancer survivors, contributing to reduced work ability and increased absenteeism [84,85]. To evaluate self-efficacy specifically within the context of post-cancer treatment management, we recommend the use of the Stanford 6-item scale for self-efficacy for managing chronic disease [86], validated with a cancer population [87]. This scale was designed to assess self-efficacy in managing common post-treatment challenges, making it highly relevant for understanding participants’ confidence in overcoming work-related obstacles during RTW efforts. Furthermore, we suggest adopting the Cancer and Work Scale (CAWSE) [1], a validated oncology-specific vocational rehabilitation tool, as a core outcome measure in future trials. The CAWSE provides a comprehensive assessment of key RTW factors, enabling healthcare providers to develop targeted interventions that enhance RTW outcomes and long-term employment sustainability for individuals transitioning back to work after cancer treatment.

### 4.3. Limitations

This study has several limitations. Notably, a key limitation of this study is the small sample size, which restricts statistical power and limits the generalizability of findings. Although we included exploratory statistical comparisons, these were conducted to examine trends that could inform the selection of a primary outcome for a future definitive trial rather than to determine efficacy. The small number of participants, particularly in the control group, and the presence of missing data further constrained our ability to conduct parametric testing to test for statistically significant differences. Therefore, all findings should be interpreted with caution. Future studies should be adequately powered to account for attrition and allow for more robust between-group comparisons of intervention effects.

Another limitation comes from our sample lacking diversity in terms of gender and cancer diagnoses. The majority of participants were women with breast cancer, many of whom were highly educated and in a relationship, hence likely having a support system, which can be a determining factor in RTW after cancer. While this population reflects a significant proportion of individuals identified as being on sick leave due to cancer, the limited diversity leaves uncertainty regarding the intervention’s acceptability and effectiveness for other groups of ITBC. Future research should aim to explore the feasibility and acceptability of iCanWork among more diverse populations, including among men, individuals with other cancer types, those with varying educational backgrounds, and people in different social circumstances.

Several practical challenges impacted this study. Funding limitations, time constraints, and difficulties in scheduling sessions compatible for both the participants and the VR and OT posed barriers to participant engagement. These logistical challenges may have affected participants’ ability to engage fully with the intervention or commit to completing this study’s post-study measures. Additionally, this study concluded before some participants had attempted RTW, limiting the opportunity to observe outcomes in those still preparing to re-enter the workforce. This may have underestimated the intervention’s potential impact on RTW outcomes.

Another limitation concerns the selection of distal work-health related outcomes. In this pilot study, one of the work-health related outcomes was assessed using binary RTW status. However, binary indicators do not fully capture the complexity and sustainability of work reintegration. In future trials, we recommend adopting more nuanced RTW outcomes, such as sustainable RTW, defined as a return to full-time or part-time work (≥50% of pre-diagnosis hours) maintained for at least 28 consecutive days, similar to the approach used by Fassier et al. [18,88,89].

Future research may also benefit from incorporating more proximal outcomes that align directly with intervention strategies (e.g., identifying and engaging in workshops on managing cancer-related fatigue at work) to better capture short-term changes resulting from the intervention. Finally, the low STTS-R [52] response rate among control group participants limits the interpretability of satisfaction findings. Moreover, the STTS-R may not be the most suitable tool for assessing satisfaction with educational resources such as a website to represent the intervention being assessed. If the goal is to evaluate whether a website effectively informs and guides participants in their RTW planning, alternative measures designed for this purpose would be more appropriate.

### 4.4. Implications for Future Research and Policy

The persistence of poor work ability scores in both groups at follow-up underscores the need for prolonged support and extended RTW interventions. Future studies should incorporate extended follow-up periods to assess long-term employment sustainability beyond initial RTW. In this context, the introduction of a partial disability program—defined as a structured, part-time, or modified return-to-work arrangement accompanied by proportional income replacement—may offer a promising strategy. Such programs allow ITBCs to gradually reintegrate into the workforce while accommodating ongoing health challenges. Drawing on successful models from Nordic countries [90], partial disability benefits have been associated with improved work attachment and reduced long-term disability risk. Research is needed to assess the feasibility, acceptability, and effectiveness of these programs within cancer survivorship care, especially in jurisdictions where occupational flexibility and cross-sector collaboration can be supported through policy development and health system integration.

Another key recommendation is a better selection of outcome measures that are more closely aligned with the iCanWork intervention potential areas of impact. While work ability remains a critical indicator, future studies should also incorporate measures that capture broader psychosocial and functional dimensions of RTW success, such as self-efficacy, autonomy, RTW readiness, knowledge of workplace accommodations, meaning of work, and financial impact. Notably, several of these concepts, such as meaning of work, autonomy, and financial impact, are already captured within specific subscales of the CAWSE [1]. These domains are often integral to the RTW process for ITBCs and could provide a more nuanced understanding of the intervention’s benefits.

Moreover, developing an AI-enhanced active control group could improve the comparability of future trials. Leveraging AI-based guidance using structured resources from the Cancer and Work website could offer scalable and interactive support for control participants. This approach would ensure a more rigorous evaluation of intervention effectiveness while maintaining ethical considerations by providing meaningful guidance to all study participants.

## 5. Conclusions

The iCanWork intervention was found to be feasible and acceptable, achieving high recruitment and retention rates, strong protocol fidelity, and positive participant feedback. Although this pilot trial was not powered to detect statistical differences, exploratory trends in RTW status, work ability, and quality of life suggest potential benefits associated with the intervention. These findings, alongside participants’ reports of increased confidence, improved communication, and better preparation for workplace reintegration, support the relevance of a theory-informed, multidisciplinary RTW approach for ITBCs.

The results of this pilot study inform both the selection of outcomes and the refinement of intervention strategies for future research. Larger, fully powered trials are now warranted to evaluate the effectiveness of iCanWork, incorporating enhanced components such as employer engagement, structured physical rehabilitation, and nuanced outcome measures, including sustained RTW and working hours. With these enhancements, iCanWork has the potential to become a comprehensive, scalable, and person-centered RTW solution for ITBCs.

## Figures and Tables

**Table 1:** Participant demographic and clinical characteristics.

Variable	iCanWork (n = 12)	Control (n = 11)	Total (n = 23)
Age (years)	Mean (SD): 43.08 ± 9.22	Mean (SD): 48.27 ± 8.34	Mean (SD): 45.57 ± 9.01
Months to RTW	Mean (SD): 9.75 ± 5.34	Mean (SD): 12.55 ± 6.56	Mean (SD): 11.09 ± 5.99
Gender			
Women	12 (100.0%)	10 (90.9%)	22 (95.7%)
Men	0 (0.0%)	1 (9.1%)	1 (4.3%)
Marital Status			
Married/Common law	10 (62.5%)	6 (37.5%)	16 (69.6%)
Never Married	2 (50.0%)	2 (50.0%)	4 (17.4%)
Separated	0 (0.0%)	2 (100.0%)	2 (8.7%)
Other	0 (0.0%)	1 (100.0%)	1 (4.3%)
Education			
Less than High School	0 (0.0%)	1 (100.0%)	1 (4.3%)
High School Graduate	1 (100.0%)	0 (0.0%)	1 (4.3%)
Some College	1 (33.3%)	2 (66.7%)	3 (13.0%)
University/College Graduate	6 (46.2%)	7 (53.8%)	13 (56.5%)
Postgraduate	4 (80.0%)	1 (20.0%)	5 (21.7%)
Diagnosis			
Breast Cancer	11 (91.7%)	8 (72.7%)	19 (82.6%)
Non-Hodgkin Lymphoma	1 (8.3%)	2 (18.2%)	3 (13.0%)
Oral Cancer	0 (0.0%)	1 (9.1%)	1 (4.3%)
Treatment			
Chemotherapy (Yes)	9 (75.0%)	9 (81.8%)	18 (78.3%)
Radiation (Yes)	5 (41.7%)	5 (45.5%)	10 (43.5%)

SD = standard deviation; RTW = return to work.

**Table 2:** Participant intervention summary VR and OT sessions.

ID	Before RTW	After RTW	Reasons
P1	1 VR	2 VR	Met the benchmark for before and after RTW
P2	2 VR, 1 OT	1 VR	Met the benchmark for before and after RTW
P3	2 VR, 1 OT	0	Met before RTW; unmet after RTW due to scheduling issues
P4	1 VR, 1 OT	1 VR	Met the benchmark for before and after RTW
P5	1 VR, 1 OT	1 VR	Met the benchmark for before and after RTW
P6	2 VR, 1 OT	0	Met before RTW; unmet after RTW due to scheduling issues
P7	2 VR, 0 OT	0	Met before RTW; OT not delivered despite attempts; RTW not attempted
P8	2 VR, 1 OT	0	Met before RTW; RTW not attempted during study time frame
P9	1 VR, 1 OT	1 VR	Met the benchmark for before and after RTW
P10	2 VR, 1 OT	0	Met before RTW; RTW not attempted during study time frame
P11	1 VR	0	Met before RTW; study ended before additional VR could occur
P12	1 VR	0	Met before RTW; study ended before additional VR could occur

Note: VR = vocational rehabilitation; OT = occupational therapy.

**Table 3:** Participant satisfaction with the iCanWork intervention and therapist (STTS-R) [52].

Domain	Item	Intervention Count Agree (%) (n = 12)	Control Count Agree (%) (n = 5)
Satisfaction with Intervention	I am satisfied with the quality of the intervention I received (Item 1).	9 (75.0%)	2 (40.0%)
	The interventionist listened to what I was trying to say (Item 2).	12 (100.0%)	2 (40.0%)
	My needs were met by the program (Item 3).	8 (66.7%)	2 (40.0%)
	The interventionist provided an adequate explanation (Item 4).	12 (100.0%)	2 (40.0%)
	I would recommend the program to a friend (Item 5).	9 (75.0%)	2 (40.0%)
	The interventionist was not negative or critical towards me (Item 6).	9 (75.0%)	2 (40.0%)
	I would return to the program if I needed help (Item 7).	9 (75.0%)	2 (40.0%)
Satisfaction with Therapist	The interventionist was friendly and warm towards me (Item 8).	12 (100.0%)	2 (40.0%)
	I am now able to deal more effectively with my problems (Item 9).	5 (41.7%)	2 (40.0%)
	I felt free to express myself (Item 10).	12 (100.0%)	2 (40.0%)
	I was able to focus on what was of real concern to me (Item 11).	10 (83.3%)	2 (40.0%)
	The interventionist seemed to understand what I was thinking and feeling (Item 12).	9 (75.0%)	2 (40.0%)
Perceived Changes in Condition	How much did this intervention help with the specific problems that led you to the program? (Item 13)	10 (83.3%)	4 (80.0%)

**Table 4:** Status and time to RTW by group allocation at three months post-baseline (T2).

RTW Status	iCanWork (n = 12)	Control (n = 11)	Total (n = 23)
Returned Full-Time	6 (50.0%)	4 (36.4%)	10 (43.5%)
Returned Part-Time	4 (33.3%)	6 (54.5%)	10 (43.5%)
Did Not Return to Work	2 (16.7%)	1 (9.1%)	3 (13.0%)
Median Time to RTW (months)	8.0	11.00	11.0
Mean Time to RTW (months)	10.4	13.5	12.1

Note: Median time to RTW reflects the time by which 50% of participants in each group returned to work. This value highlights the central tendency of RTW. Mean time to RTW indicates the average time to RTW for each group. The mean provides additional context but may be influenced by outliers.

**Table 5:** Work ability categories at T1 and T2.

Time	WAI Category	iCanWork (n, %)	Control (n, %)	Total (n)	Mann–Whitney U	Exact *p*-Value
T1	Poor (7–27 points)	4 (44.4%)	5 (55.6%)	9		
	Moderate (28–36 points)	7 (58.3%)	5 (41.7%)	12		
	Good (37–43 points)	1 (50.0%)	1 (50.0%)	2	69.50	0.33
T2	Poor (7–27 points)	5 (71.4%)	2 (28.6%)	7		
	Moderate (28–36 points)	5 (55.6%)	4 (44.4%)	9		
	Good (37–43 points)	2 (66.7%)	1 (33.3%)	3	36.50	0.650

Note: WAI = work ability index.

**Table 6:** PROPr QoL domains.

Domain	Groups n = 12 iCanWork n = 7 Control	Baseline Mean (SD) (T1)	Three Months After Baseline (T2) Mean (SD)	*p*-Value (Within Group)	*p*-Value (Between Groups)
Physical function	iCanWork	50.93 ± 7.845	50.89 ± 7.103	0.799	
	Control	42.95 ± 6.783	44.31 ± 5.818	0.116	0.995
Anxiety	iCanWork	58.75 ± 7.30	57.25 ± 6.70	0.423	
	Control	59.21 ± 8.78	60.71 ± 9.78	0.753	0.574
Depression	iCanWork	51.70 ± 8.05	53.44 ± 6.95	0.423	
	Control	54.75 ± 9.58	59.11 ± 9.91	0.753	0.280
Fatigue	iCanWork	52.23 ± 10.55	49.82 ± 5.57	0.350	
	Control	58.57 ± 8.42	58.44 ± 8.10	0.753	0.040 *
Sleep disturbance	iCanWork	51.05 ± 10.90	47.92 ± 10.61	0.173	
	Control	58.66 ± 9.64	55.67 ± 8.38	0.225	0.131
Social roles and activities	iCanWork	51.61 ± 1.39	51.61 ± 1.39	0.262	
	Control	44.54 ± 6.67	43.63 ± 6.14	0.917	0.017 *
Pain interference	iCanWork	48.78 ± 8.098	49.39 ± 8.249	0.735	
	Control	58.14 ± 10.237	61.47 ± 6.265	0.893	0.006 *
Pain intensity	iCanWork	2.70 (3.03)	1.83 (2.41)	0.262	
	Control	3.80 (2.52)	5.43 (2.15)	0.202	0.063

* *p* = < 0.05 (significance levels between the adjusted mean scores of the control and experimental groups after intervention).

## Data Availability

The data presented in this study are available upon reasonable request from the corresponding author. Due to the sensitive nature of health and vocational implications, as well as the potential risks of bias and stigmatization, access to the data is restricted.

## Supplementary Materials

The following supporting information can be downloaded at https://www.mdpi.com/article/10.3390/curroncol32050266/s1, Table S1: CONSORT 2010 checklist of information to include when reporting a pilot or feasibility trial. Table S2: The template for intervention description and replication (TIDieR) checklist. Table S3: Evaluation grid for the iCanWork RTW program for ITBCs. Supplementary Document S4: Examples of intake sessions and summary notes from the VR and OT hired for this study.

## Abbreviations

The following abbreviations are used in this manuscript:

AI	Artificial Intelligence
ANOVA	Analysis of Variance
CAWSE	Cancer and Work Scale
CFIR	Consolidated Framework for Implementation Research
CONSORT	Consolidated Standards of Reporting Trials
ILWC	Individuals Living with Cancer
ITBC	Individuals Touched by Cancer
NOC	National Occupational Classification
NPT	Normalization Process Theory
OT	Occupational Therapy
PROM	Patient-Reported Outcome Measure
QoL	Quality of Life
RCT	Randomized Controlled Trial
RTW	Return to Work
STTS-R	Satisfaction with Therapy and Therapist Scale-Revised
TIDieR	Template for Intervention Description and Replication
VR	Vocational Rehabilitation
WAI	Work Ability Index
