# Supplementary material for: Pilot Randomized Controlled Trial of iCanWork: Theory-Guided Return-to-Work Intervention for Individuals Touched by Cancer
Source: Curr Oncol. 2025 May 1;32(5):266. doi: 10.3390/curroncol32050266 (PMC12110094; doi:10.3390/curroncol32050266)
Supplement: Supplementary file 1 [file curroncol-32-00266-s001.zip › curroncol-3497466-supplementary.pdf]

Table S1 *CONSORT 2010 checklist of information to include when reporting a pilot or feasibility trial*

Section/ Topic	Item No	Checklist item	Reported on page #/ line #/ section *
**Title and abstract**			
	1a	Identification as a pilot or feasibility randomised trial in the title	title (p1)
	1b	Structured summary of pilot trial design, methods, results, and conclusions (for specific guidance see CONSORT abstract extension for pilot trials)	p1, line 21-42
**Introduction**			
Background and objectives	2a	Scientific background and explanation of rationale for future definitive trial, and reasons for randomised pilot trial	line 47-160
	2b	Specific objectives or research questions for pilot trial	line 159-163
**Methods**			
Trial design	3a	Description of pilot trial design (such as parallel, factorial) including allocation ratio	line 165
	3b	Important changes to methods after pilot trial commencement (such as eligibility criteria), with reasons	NA
Participants	4a	Eligibility criteria for participants	line 263-272
	4b	Settings and locations where the data were collected	line 350-352
	4c	How participants were identified and consented	line 283-286
Interventions	5	The interventions for each group with sufficient details to allow replication, including how and when they were actually administered	section 2.1, line 165-253
Outcomes	6a	Completely defined prespecified assessments or measurements to address each pilot trial objective specified in 2b, including how and when they were assessed	section 2.6, line 372-446
	6b	Any changes to pilot trial assessments or measurements after the pilot trial commenced, with reasons	NA
	6c	If applicable, prespecified criteria used to judge whether, or how, to proceed with future definitive trial	section 2.6, line 372-386
Sample size	7a	Rationale for numbers in the pilot trial	line 273-282
	7b	When applicable, explanation of any interim analyses and stopping guidelines	N/A
Randomisation:			
Sequence generation	8a	Method used to generate the random allocation sequence	line 283-296
	8b	Type of randomisation(s); details of any restriction (such as blocking and block size)	line 290
Allocation, concealment mechanism	9	Mechanism used to implement the random allocation sequence (such as sequentially numbered containers), describing any steps taken to conceal the sequence until interventions were assigned	line 283-296
Implementation	10	Who generated the random allocation sequence, who enrolled participants, and who assigned participants to interventions	line 283-296
Blinding	11a	If done, who was blinded after assignment to interventions (for example, participants, care providers, those assessing outcomes) and how	line 293-296
	11b	If relevant, description of the similarity of interventions	N/A
Statistical methods	12	Methods used to address each pilot trial objective whether qualitative or quantitative	section 2.7, line 447-475

**Results**			
Participant flow (a diagram is strongly recommended)	13a	For each group, the numbers of participants who were approached and/or assessed for eligibility, randomly assigned, received intended treatment, and were assessed for each objective	Figure 3, p 10, line 486-490
	13b	For each group, losses and exclusions after randomisation, together with reasons	Figure 3, p 11
Recruitment	14a	Dates defining the periods of recruitment and follow-up	line 261-262
	14b	Why the pilot trial ended or was stopped	N/A
Baseline data	15	A table showing baseline demographic and clinical characteristics for each group	Table 1, p 11-12
Numbers analysed	16	For each objective, number of participants (denominator) included in each analysis. If relevant, these numbers should be by randomised group	Figure 3, p 11
Outcomes and estimation	17	For each objective, results including expressions of uncertainty (such as 95% confidence interval) for any estimates. If relevant, these results should be by randomised group	Tables 4-6
Ancillary analyses	18	Results of any other analyses performed that could be used to inform the future definitive trial	sections 3.2-3.3, Tables 3, p 14
Harms	19	All important harms or unintended effects in each group (for specific guidance see CONSORT for harms)	NA
	19a	If relevant, other important unintended consequences	NA
**Discussion**			
Limitations	20	Pilot trial limitations, addressing sources of potential bias and remaining uncertainty about feasibility	line 772-816
Generalisability	21	Generalisability (applicability) of pilot trial methods and findings to future definitive trial and other studies	line 684-699
Interpretation	22	Interpretation consistent with pilot trial objectives and findings, balancing potential benefits and harms, and considering other relevant evidence	line 629-652
	22a	Implications for progression from pilot to future definitive trial, including any proposed amendments	line 808-837
**Other information**			
Registration	23	Registration number for pilot trial and name of trial registry	NA
Protocol	24	Where the pilot trial protocol can be accessed, if available	NA
Funding	25	Sources of funding and other support (such as supply of drugs), role of funders	line 876
Ethical approval	26	Ethical approval or approval by research review committee, confirmed with reference number	line 174, 881

Reference: Eldridge SM, Chan CL, Campbell MJ, Bond CM, Hopewell S, Thabane L, et al. CONSORT 2010 statement: extension to randomised pilot and feasibility trials. BMJ. 2016;355. This is an Open Access article distributed in accordance with the terms of the Creative Commons Attribution (CC BY 3.0) license (http://creativecommons.org/licenses/by/3.0/), which permits others to distribute, remix, adapt and build upon this work, for commercial use, provided the original work is properly cited.

Table S2 *The template for intervention description and replication (TIDieR) checklist*

Item	Item	Where located *	
		Primary paper	Other † (details)
	**BRIEF NAME**		
**1.**	Provide the name or a phrase that describes the intervention.	Title, p1	
	**WHY**		
**2.**	Describe any rationale, theory, or goal of the elements essential to the intervention.	Introduction, line 48-163	
	**WHAT**		
**3.**	Materials: Describe any physical or informational materials used in the intervention, including those provided to participants or used in intervention delivery or in training of intervention providers. Provide information on where the materials can be accessed (e.g. online appendix, URL).	line 164–253; 296-346; Suppl. S3	
**4.**	Procedures: Describe each of the procedures, activities, and/or processes used in the intervention, including any enabling or support activities.	section 2.1, line 166–255	
	**WHO PROVIDED**		
**5.**	For each category of intervention provider (e.g. psychologist, nursing assistant), describe their expertise, background and any specific training given.	line 294-305; Suppl. 4	
	**HOW**		
**6.**	Describe the modes of delivery (e.g. face-to-face or by some other mechanism, such as internet or telephone) of the intervention and whether it was provided individually or in a group.	line 298-309	
	**WHERE**		
**7.**	Describe the type(s) of location(s) where the intervention occurred, including any necessary infrastructure or relevant features.	NA (virtual)	
	**WHEN and HOW MUCH**		
**8.**	Describe the number of times the intervention was delivered and over what period of time including the number of sessions, their schedule, and their duration, intensity or dose.	line 311-315	
	**TAILORING**		
**9.**	If the intervention was planned to be personalised, titrated or adapted, then describe what, why, when, and how.	line 175-182	
	**MODIFICATIONS**		
**10.**[‡]	If the intervention was modified during the course of the study, describe the changes (what, why, when, and how).	N/A	

Item	Item	Where located *	
		Primary paper	Other † (details)
	**BRIEF NAME**		
**1.**	Provide the name or a phrase that describes the intervention.	Title, p1	
	**HOW WELL**		
**11.**	Planned: If intervention adherence or fidelity was assessed, describe how and by whom, and if any strategies were used to maintain or improve fidelity, describe them.	line 369-371; 373-392	
**12.ǂ**	Actual: If intervention adherence or fidelity was assessed, describe the extent to which the intervention was delivered as planned.	section 3.2, line 498-529	

† If the information is not provided in the primary paper, give details of where this information is available. This may include locations such as a published protocol or other published papers (provide citation details) or a website (provide the URL).ǂ If completing the TIDieR checklist for a protocol, these items are not relevant to the protocol and cannot be described until the study is complete.

Table S3 *Evaluation Grid for the iCanWork RTW Program for ITBC.*

**4 Factor VR Model for ITBC** 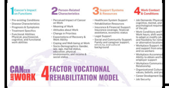	**Criterion**	**Initial Assessment (IA)** [a] **by VR**	**Follow-up 1** [b] **by VR and/or OT**	**Follow-up 2** [c] **by VR and/or OT**	**Follow-up 3** [d] **by VR and/or OT**	**Example Thesaurus** [e]
**1) Cancer's Impact on Functions**	Pre-existing conditions<br>Disease Characteristics<br>Treatment Specifics	High<br>Moderate<br>Low	High Moderate Low	High Moderate Low	High Moderate Low	IA: Assess for chronic conditions (e.g., cardiovascular, diabetes, musculoskeletal issues) that may affect fatigue, endurance, or physical ability, and develop a personalized **Evaluation Summary FU1:**<br>FU2:<br>FU3:
	Physical Function	High<br>Moderate<br>Low	High Moderate Low	High Moderate Low	High Moderate Low	IA: Assess functions and consider using tools:<br>Assessment tools: WAI, WMS, OCFQ, etc.<br>Review ergonomic adaptations<br>Evaluation Summary FU1 / FU2 / FU3
	Cognitive Function	High<br>Moderate<br>Low	High Moderate Low	High Moderate Low	High Moderate Low	IA: Follow up needed in neurocognitive rehabilitation<br>Evaluation Summary FU1 / FU2 / FU3
	Psychological Function	High<br>Moderate<br>Low	High Moderate Low	High Moderate Low	High Moderate Low	IA: Continuous psychological support required<br>Evaluation Summary FU1 / FU2 / FU3
**2) Person Related Factors**	Attitudes towards Work<br>Perceived Self-Efficacy<br>Meaning of Work<br>Change in Priorities	High<br>Moderate<br>Low	High Moderate Low	High Moderate Low	High Moderate Low	IA: Strong motivation and engagement<br>Evaluation Summary FU1 / FU2 / FU3

	Socio demographic Influences	High<br>Moderate<br>Low	High Moderate<br>Low	High Moderate<br>Low	High Moderate<br>Low	IA: Identify socio-demographic barriers and strengths.<br>FU1: Introduce tailored vocational resources (e.g., training program for digital literacy)<br>Evaluation Summary FU1 / FU2 / FU3
**3) Support Systems & Resources**	Access to Healthcare Services<br><br>Rehabilitation Resources	High<br>Moderate<br>Low	High Moderate<br>Low	High Moderate<br>Low	High Moderate<br>Low	IA: Improve healthcare support and Improve access to rehabilitation services<br>Evaluation Summary FU1 / FU2 / FU3
	Social and Community Support	High<br>Moderate<br>Low	High Moderate<br>Low	High Moderate<br>Low	High Moderate<br>Low	IA: Strengthen family support<br>Implement specific support measures<br>Evaluation Summary FU1 / FU2 / FU3
	Insurance Support	High<br>Moderate<br>Low	High Moderate<br>Low	High Moderate<br>Low	High Moderate<br>Low	IA: Improve insurance coverage<br>Evaluation Summary FU1 / FU2 / FU3
**4) Work Context and Conditions**	Job Demands<br>Work Conditions and Flexibility	High<br>Moderate<br>Low	High Moderate<br>Low	High Moderate<br>Low	High Moderate<br>Low	IA:<br>Evaluation Summary FU1 / FU2 / FU3
	Workplace Accommodations<br>Workplace Support	High<br>Moderate<br>Low	High Moderate<br>Low	High Moderate<br>Low	High Moderate<br>Low	IA: Adequate adaptations; Awareness and staff training<br>Evaluation Summary FU1 / FU2 / FU3

a At each assessment phase, participants' likelihood to RTW and sustain employment is assessed using the CAWSE scale (Maheu et al. 2025). Participants scoring on the lower end of the scale are identified as facing greater barriers to RTW and sustain employment. Specific CAWSE items with low scores are used to pinpoint individual challenges that may be contributing to their placement in the lower likelihood category. Tailored interventions are designed to address these obstacles.
b FUI: Identify barriers contributing to lower CAWSE scores and tailor interventions such as fatigue management strategies, ergonomic adjustments, or referrals to specialists.
c FU2: Evaluate progress and adjust intervention intensity based on improved or persisting barriers.
d FU3: Assess likelihood for sustained employment and ensure long-term strategies are in place to maintain workplace success.
e Tailored strategies vary by CAWSE score to address individual barriers.

Supplemental S4 - Illustrative Documentation from iCanWork Sessions

This supplemental file presents illustrative documentation from iCanWork intervention sessions, showcasing how clinical notes were recorded by vocational rehabilitation counsellors and occupational therapists. These examples highlight the individualized and structured nature of session planning, delivery, and follow-up.

Section 1: Vocational Rehabilitation Counsellor (VRC) Intake Session Overview and Summary

Participant Overview: The participant is a 35 year-old professional who has been on medical leave following a diagnosis of Stage 3 breast cancer. She underwent comprehensive cancer treatment, including chemotherapy, surgery, radiotherapy, and immunotherapy. Currently, she is preparing for a graduated RTW as a nurse practitioner, starting part-time at her clinic. Despite her motivation and eagerness to return, she reports lingering challenges such as fatigue, cognitive difficulties ("brain fog"), and balancing her professional and personal responsibilities, including managing her young child and household tasks.

Health and Psychosocial Background: The participant is in remission but continues targeted hormonal therapy and experiences mild anxiety and cognitive difficulties. She has a history of effective psychotherapy and self-initiated coping mechanisms but paused sessions during active treatment. She utilizes tools learned in therapy to manage stress and seeks additional strategies to support her transition back to work. Physical challenges include shoulder stiffness post-mastectomy, which she addresses through physiotherapy and osteopathy.

Work and Personal Challenges: The participant reports fatigue and cognitive difficulties that could affect her performance in a fast-paced, multitasking role. At home, she has taken on additional responsibilities due to her extended medical leave but is now seeking to delegate more tasks, particularly childcare. She expresses concerns about maintaining her work-life balance and effectively managing her workload.

Current Strengths and Motivation: The participant demonstrates high motivation, a positive mindset, and a strong commitment to returning to her career, which she values deeply. She has implemented structured organizational strategies, such as calendars and task lists, to help manage daily activities. Her support system includes her partner, who has adjusted his work schedule to assist at home.

Recommendations Summary:

1. **Cognitive and Emotional Support:** Encourage the participant to speak with her family doctor about cognitive-behavioral therapy (CBT) for anxiety and cognitive challenges. Journaling was recommended to help track mood and energy levels to identify patterns and triggers.
2. **Workplace Accommodations:** Provide resources on workplace accommodations and RTW planning, including communication strategies with her employer and healthcare providers. Suggested collaboration with an occupational therapist (OT) for tailored stress and anxiety management techniques.
3. **Mindfulness and Relaxation:** Recommend mindfulness and meditation resources, such as guided programs available online (e.g., Petit Bambou app, Palouse Mindfulness). Introduce tailored programs like Wellspring's brain fog and coaching sessions.
4. **Community Resources:** Highlight local support services, such as the Quebec Cancer Foundation, for additional in-person resources and networking.
5. **Work-Life Balance Strategies:** Encourage the participant to delegate household responsibilities and manage her workload to align with her energy levels. Suggest evaluating her work tasks and prioritizing those requiring higher cognitive demand in the morning when her energy is better.

Follow-Up: The participant was provided with Cancer and Work resources, including tools for RTW planning, workplace accommodations, and stress management. A connection with an internal OT was recommended for further assistance.

Section 2: Occupational Therapist's Intake Session Overview and Summary

Participant Overview
A middle-aged individual diagnosed with stage III non-Hodgkin's lymphoma in late 2021 underwent chemotherapy and radiation therapy, concluding treatment in 2022. Post-treatment complications included jaundice, metabolic changes, endocrine dysregulation (cortisol imbalance), and a diagnosis of osteoarthritis in 2022, causing significant musculoskeletal pain, which has been managed through a combination of therapy and cortisone injections. Additional health considerations include thyroid dysfunction and menopause-related symptoms.

Functionality and Current Status

- **Activities of Daily Living (ADLs)**: Independent, including managing household tasks and transportation.
- **Leisure Activities**: Enjoys socializing, walking, and engaging in regular physical activity.
- **Work**: Preparing to start a new professional role in a remote capacity. Feels optimistic but reports concerns about maintaining energy and managing cognitive challenges.
- **Energy and Cognitive Concerns**: Experiences reduced energy levels in the afternoons and subjective cognitive difficulties, such as word-finding issues, multitasking challenges, and occasional forgetfulness.

Recommendations for Return to Work

1. **Energy Conservation and Managing Cognitive Symptoms**:
    - Implement regular short breaks throughout the workday for stretching and mindfulness exercises.
    - Incorporate relaxation or short rest periods during midday breaks.
    - Prioritize complex tasks during high-energy periods, typically in the morning.
    - Minimize multitasking by focusing on one task at a time and maintaining a structured work schedule.
    - Continue engaging in regular physical activity to support energy and cognitive function.
2. **Ergonomic Workstation Setup**:
    - Adhere to ergonomic best practices for remote workstations, including optimal posture and equipment positioning.
    - Utilize resources from relevant workplace accommodation guides.
    - Discuss with the employer the possibility of a formal ergonomic evaluation.
3. **Psychosocial and Emotional Support**:
    - Explore counseling or cognitive-behavioral therapy to address anxiety and residual stress related to the cancer experience.
    - Consider additional support for processing the psychosocial impacts of cancer survivorship.

Email Summary Sent to Participant
The occupational therapist prepared and sent a detailed written summary within 24 hours, including:

- Tailored strategies for managing energy and cognitive symptoms.
- Guidance on ergonomic workstation setup.
- Recommendations for emotional and coping support resources.

This session illustrates a holistic and personalized approach to supporting the participant's return to work, emphasizing resilience and adaptability in the face of physical and cognitive challenges.

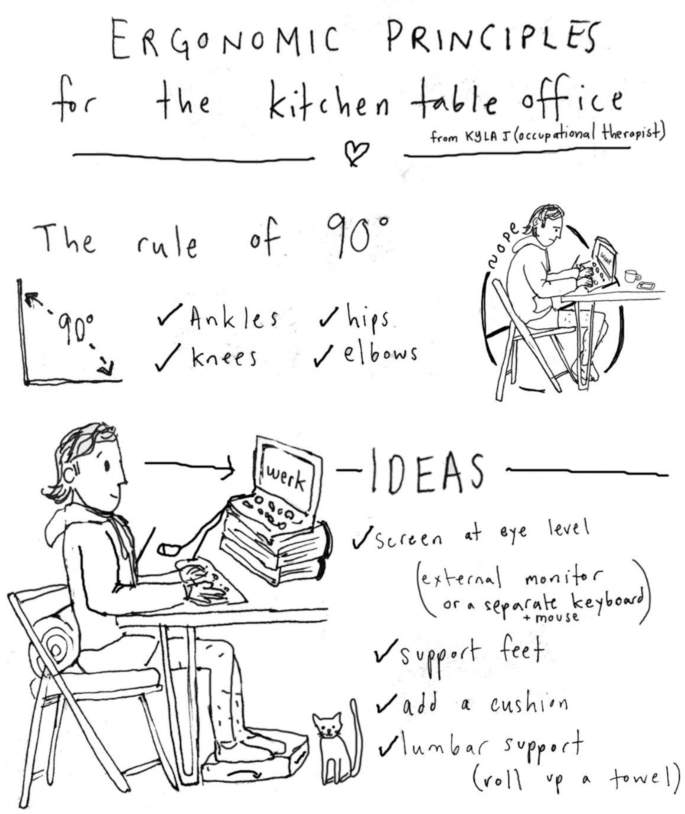

Section 3: VRC Session #2 for a study participant

Medical Background:

- Breast cancer diagnosis June 2020 followed by surgery, radiation, and chemotherapy. Treatment completed June 2021.
- Preventative surgery scheduled for August 25, 2022, to remove ovaries and uterus due to a family history of ovarian cancer (mother and sister affected).
- Currently under surveillance for ovarian risk, no cancer diagnosis.
- Sick leave extended from July 2022 to October 2022 for post-surgical recovery.

Bio-Psychosocial Considerations:

- The participant was informed this type of surgery typically requires six weeks for recovery. However, her RTW date is set for October 1st, allowing only five weeks post-surgery.
- She feels pressured to return due to nearing the two-year mark for her disability claim.
- The vocational rehabilitation counsellor (VRC) explained sick leave policies and the definition of "own occupation disability" within disability case management. Advocacy strategies with healthcare providers and case managers were discussed.
- The participant expressed concerns about her capacity to sustain work due to persistent fatigue and the need for frequent naps.

Recommendations for Post-Surgery Recovery and RTW Planning:

- The VRC advised her to consult with her physician for a rationale to extend her RTW date to align with the standard recovery period and submit the necessary documentation to her disability case manager.
- To address fatigue and functional capacity concerns, the VRC suggested:
    - Speaking with her doctor about a referral to a "work hardening program" or "rehabilitation activation program," involving occupational and physical therapy.
    - Identifying rehabilitation clinics in her area with these programs. Kinatex, with multiple locations in Montreal, was recommended.
    - Consulting a physiotherapist to support physical recovery.
- Volunteering during her extended leave was suggested to help build stamina and maintain engagement.

Psychosocial and Cognitive Concerns:

- The participant expressed relief after an assessment by the study OT, who strategies to support her recovery.
- The participant is actively working on personal development, including improving her English and addressing health concerns.
- The VRC highlighted the challenges of prolonged absence from work and emphasized the importance of proactive planning for a successful RTW.

Resources Provided:

- RTW planning tools and questions from the Cancer and Work website: Develop a Return-to-Work Plan
- Educational resources on navigating RTW.

Permission letter for figure 1 previously published

International Foundation OF EMPLOYEE BENEFIT PLANS
Education | Research | Leadership

TRANSMITTED VIA E-MAIL
christine.maheu@mcgill.ca

March 18, 2025

Christine Maheu
McGill University
845 Sherbrooke Street West
Montréal, QC H3A 0G4

Dear Ms. Maheu:

The International Foundation of Employee Benefit Plans grants you permission to publish the "Four Factor Vocational Rehabilitation Model" that appeared in the November/December 2024 issue of *Plans & Trusts* in the journal *iCanWork*. If the exact same image will be used, include the citation of "@ 2025 International Foundation of Employee Benefit Plans. All rights reserved." with the image. Also please provide a full citation to the *Plans & Trusts* issue in the endnotes.

Sincerely,

Cathe Gooding

Cathe Gooding
Director
Research and Publications

CG/as

18700 West Bluemound Road, Brookfield, WI 53045
Telephone (262) 786-6700 | Fax (262) 786-8670
www.ifebp.org
